# The Influence of the Degree of Dental Implant Insertion Compression on Primary Stability Measured by Resonance Frequency and Progressive Insertion Torque: In Vitro Study

**DOI:** 10.3390/biomedicines12122878

**Published:** 2024-12-18

**Authors:** José Rosas-Díaz, Maria Eugenia Guerrero, Nancy Córdova-Limaylla, Maisely Galindo-Gómez, Marco García-Luna, César Cayo-Rojas

**Affiliations:** 1 School of Stomatology, Universidad Privada San Juan Bautista, Lima 15067, Peru; nancye.cordova@upsjb.edu.pe (N.C.-L.); maisely.galindo@upsjb.edu.pe (M.G.-G.); marco.garcial@upsjb.edu.pe (M.G.-L.); 2 Faculty of Stomatology, Universidad Peruana Cayetano Heredia, Lima 15102, Peru; 3 Medico Surgical Department, Faculty of Dentistry, Universidad Nacional Mayor de San Marcos, Lima 15081, Peru; mguerreroac@unmsm.edu.pe

**Keywords:** implant stability, dental implants, resonance frequency analysis

## Abstract

**Background**: This study aimed to evaluate the primary stability, according to the insertion torque value (ITV) and resonance frequency analysis (RFA), of dental implants placed in standardized blocks of bone quality equivalent to type II-A bone, using three surgical undersized protocols of 0.2 mm, 0.5 mm, and 0.8 mm, considering different dental implant diameters and lengths. **Methods**: One hundred and twenty dental implants (DIs) of different diameters (3.5, 3.8, 4.5, and 5.0 mm) and lengths (8.5, 10.0, 11.5, 13.0, and 15.0 mm) placed in polyurethane blocks equivalent to type II-A bone, according to the Lekholm and Zarb classification modified by Rosas et al., were examined with three surgical protocols of under-milling of 0.2, 0.5, and 0.8 mm. The ITV and the RFA were the determinants of primary stability, and their respective values were recorded as Ncm and the implant stability quotient (ISQ) immediately after the placement of the DIs. These were evaluated according to each surgical insertion protocol, length, and diameter of the DI under a multivariate analysis model (MANOVA). Statistical significance was set at *p* < 0.05. **Results**: It was observed that the average of the ITV was significantly higher when a 0.8 mm under-milling protocol was used (63.2 ± 14.9 Ncm) (*p* < 0.001). However, the ITV was significantly lower when a 0.2 mm under-milling protocol was used (25.1 ± 8.3 Ncm) (*p* < 0.001). On the other hand, the ISQ did not present significant differences (*p* = 0.166) when comparing the 0.2 (67.6 ISQ ± 5.4 ISQ), 0.5 (65.8 ISQ ± 3.4 ISQ), and 0.8 (65.7 ISQ ± 4.0 ISQ) under-milling protocols in the evaluation of the primary stability of the dental implant. The multivariate effect size (η*p*^2^ = 0.639) indicated that the variability detected in the insertion torque and the ISQ, at the same time, was explained by 63.9% (*p* < 0.001) due only to the compression protocol, while the implant diameter explained this variability by 27.0% (η*p*^2^ = 0.270) (*p* < 0.001) and the implant length only significantly explained this variability by 12.1% (η*p*^2^ = 0.121) (*p* = 0.030). Finally, any interaction between the compression protocol, implant diameter, and length did not influence insertion torque variability or the ISQ (*p* > 0.05). **Conclusions**: It can be concluded that when the surgical protocol for subpreparation is optimal according to the prepared bone bed, regardless of the diameter or length of the dental implant used, primary stability was assured according to the ITV and the RFA in 63.9%. This finding allows us to recommend carrying out a correct analysis of bone quality in order to subsequently select the most appropriate surgical protocol for the subpreparation of the bone bed to achieve better primary stability of the dental implant.

## 1. Introduction

Dental implant treatment has increased in recent years. A historical analysis of dental implantology leads us to understand that the surgical protocol used was extrapolated from animal studies but never derived from experimental comparative studies [1]. Empirically, Branemark et al. initially proposed a protocol of a 0.75 mm difference between the prepared bone bed and the final diameter of the dental implants. This has now been modified, with values ranging from 0 mm to 1 mm of difference being found and various surgical protocols developed with different degrees of bone compression [2]. The concept of an undersized surgical protocol is not new in implantology [3]; research has been driven by other studies using various compressive protocols to achieve greater primary stability in implants subjected to immediate loading [4], finding success rates very similar to those reported for conventionally loaded implants [5]. This seems to indicate that compressive under-milling protocols can improve primary stability by increasing torque values measured in Newtons (N) for conventional and immediate loading [6,7]. However, it should be taken into account that the application of high levels of insertion torque could damage the geometric shape of the connection depending on the physical deformation properties of the type of titanium used by each commercial company, as this could cause future problems in the rehabilitation stage, generating frequent complications such as the maladjustment of the prosthetic screws [8].

Currently, it seems that the clinician’s goal is to speed up the loading time of dental implants without decreasing success rates [9]. There is a lot of literature that supports the achievement and improvement of primary stability in splinted implants using rigid structures in immediate loads [10,11], but there is little information relating primary stability to the length, the diameter of dental implants (DIs), bone quality, and the insertion torque value obtained according to the surgical protocol used [12]. Furthermore, it would be useful to understand how the degree of undersize influences the achievement of primary stability of DIs in non-splinted single pieces, since this is the most frequent indication in current dental implantology. It is believed that the most important determinant of DI success is the achievement of excellent primary stability within other variables [13,14]. Therefore, it is of utmost importance to be able to analyze the stability of the DI during its placement to reduce dental implant complications. It is of great interest to know the variables that modify it, among which the surgical protocol stands out according to the degree of compression as one of the most important causes that, associated with the bone quality and the macro design of the DI, results in the final mechanical stability of the implant.

There are two well-recognized objective and quantitative methods to assess the primary stability of the DI, and they are resonance frequency analysis (RFA) and the insertion torque value (ITV). RFA allows us to measure in a clinical and non-invasive way the rigidity of the implant inside the bone; it is very useful to evaluate the osseointegration curve and the final osseointegration of the DI [15,16]. The ITV allows us to perform a more objective and quantifiable evaluation of the mechanical locking of the dental implant about bone quality [17]. The disadvantage of using the ITV is that it cannot be used to evaluate the osseointegration curve, since it would put at risk the stability of the implants in their bone mineralization stage [18,19]. Both assessments together provide the clinician with a better understanding of primary and secondary stability until osseointegration is achieved [20].

Therefore, the aim of this in vitro study was to evaluate the primary stability according to the ITV and RFA of dental implants of different lengths and diameters placed in standardized models of bone quality equivalent to a type II-A bone, using three surgical protocols for dental implant insertion with three different undersized degrees of bone of 0.2 mm, 0.5 mm, and 0.8 mm, considering different dental implant diameters and lengths. The null hypothesis was that there were no significant changes in the primary stability according to the ITV and RFA of dental implants placed in standardized blocks of bone quality equivalent to a type II-A bone when using three undersized surgical protocols of 0.2 mm, 0.5 mm, and 0.8 mm with different diameters and lengths of the dental implants.

## 2. Materials and Methods

### 2.1. Study Design

This in vitro experimental study was carried out at the School of Dentistry of San Juan Bautista Private University between the months of January and April 2023. This study was exempted from review by the Institutional Research Ethics Committee with letter no.1478-2022-CIE-UPSJBon, 29 September 2022. In addition, this study considered the CRIS guideline (Checklist for Reporting In vitro Studies) [21].

### 2.2. Sample Size

The minimum total sample size (n = 120) was calculated based on the formula for multivariate analysis of variance (MANOVA) with 60 groups (3 types of compressive protocol × 4 types of diameters × 5 types of lengths) and two dependent variables in the statistical software G*Power version 3.1.9.7, considering a significance level (α) = 0.05 and a statistical power (1 − β) = 0.80, with an effect size f^2^ (V) = 2.69. The polyurethane blocks were randomly divided to form the groups with n = 40, according to the insertion protocol.

### 2.3. Standardized Bone Quality Models and Implants

**Figure 1:**
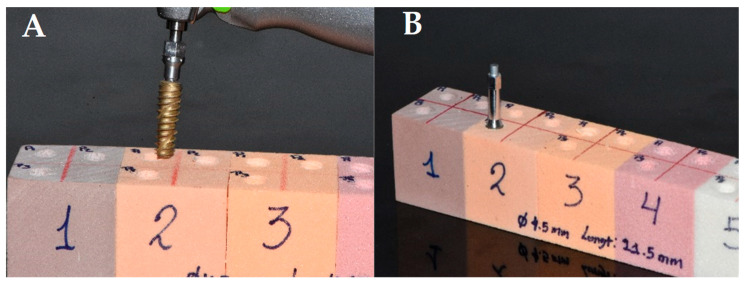
(**A**) The placement of the dental implant in the polyurethane block simulating cancellous bone. (**B**) The placement of the smartpeg to determine the primary stability of the dental implant using resonance frequency analysis.

Rectangular blocks of laminated solid rigid polyurethane foam (170 × 120 × 42 mm), simulating cancellous bone (Nacional Ossos, São Paulo, Brazil), were used to simulate type II-A bone density. Since the mean bone mineral density for the posterior maxilla is 0.31 g/cm^3^ and for the anterior maxilla is 0.55 g/cm^3^, 20 blocks of polyurethane foam of 0.32 g/cm^3^ to 0.48 g/cm^3^ were used to simulate medium-density cancellous bone (Figure 1). The implant beds in the blocks were prepared by a single operator with proven experience (JRD), who made 120 perforations following the drilling protocol recommended by the manufacturer. The dental implants were thus prepared (SIN—Epikut diameters 3.5, 3.8, 4.5, and 5.0 mm × 8.5, 10.0, 11.5, 13.0, and 15.0 mm in length, São Paulo, Brazil) (Figure 1). All implants had conical walls originally designed to be placed with three surgical protocols from least to greatest bone compressiveness (0.2 mm, 0.5 mm, and 0.8 mm). Each implant was placed in the standardized bone quality models following the three protocols. Each perforation was separated by 5 mm.

### 2.4. Surgical Protocols and Measurement of ITV and RFA

Since the drilling protocol affects the measurement of the ITV (Ncm) and RFA (ISQ) of the implant, the surgical protocols for the different implants placed in the test blocks followed the manufacturer’s recommendations. The 2 mm diameter drill bit was worked at 1200 RPM, and the 2.7, 3.0, 3.3, 3.6, 4.0, 4.3, and 4.8 mm drill bits at 800 RPM; the drilling length was 8.5, 10.0, 11.5, 13.0, and 15.0 mm in length. For the first under-milling protocol, the bed was prepared, leaving a difference of 0.8 mm between the last drill bit and the final diameter of the dental implant. For the intermediate protocol, it was left at 0.5 mm, and for the third protocol, it was left at 0.2 mm.

During implant insertion, the ITV was recorded in Ncm, and the ISQ was used for the RFA. The ITV measurements were performed from the first contact of the DI with the bone simulation block until its complete seating, recording the final insertion torque value in Ncm. For this purpose, a digital dynamometric torque wrench (Implantmed, W&H, Bürmoos, Austria) was used. The RFA was measured using the ISQ (Osstell Implantmed, W&H, Austria). For each measurement, three ISQ values were recorded, and the repeated value was used.

### 2.5. Statistical Analysis

All data were imported using SPSS (Statistical Package for the Social Sciences Inc. IBM, Armonk, NY, USA) version 28.0. For descriptive statistics, central tendency and dispersion measures such as the mean and standard deviation were used. To test the hypothesis, the normal distribution and homoscedasticity of the data were assessed using the Shapiro–Wilk and Levene tests, respectively. When the statistical requirements were met, the decision was made to use Welch’s robust ANOVA (analysis of variance) with an intergroup factor and Tukey’s post hoc. In addition, the Wilks’ Lambda MANOVA (multivariate analysis of variance) test was used with two dependent variables, and the partial Eta squared (η*p*^2^) was used to measure the effect size. Statistical significance was set at *p* < 0.05.

## 3. Results

In the standardized model of bone quality equivalent to a type II-A bone according to the Lekholm and Zarb classification modified by Rosas et al., where implants of different lengths and diameters were placed, it was observed according to the analysis of variance that the significantly highest average ITV was the one where a compressive protocol of 0.8 was used (63.2 Ncm ± 14.9 Ncm) (*p* < 0.001), regardless of the length or diameter of the DI. On the other hand, the significantly lowest ITV was with a compressive protocol of 0.2 (25.1 Ncm ± 8.3 Ncm) (*p* < 0.001). In relation to the ISQ, this did not present significant differences (*p* = 0.166) when comparing the compressive protocols of 0.2 (67.6 ISQ ± 5.4 ISQ), 0.5 (65.8 ISQ ± 3.4 ISQ), and 0.8 (65.7 ISQ ± 4.0 ISQ) in the evaluation of the primary stability of the dental implant (Table 1).

According to the full factor analysis, it was observed under the analysis of variance that the degree of bone compression according to the surgical protocol used significantly influenced primary stability, and according to the univariate analysis, as the compression protocol increased, the insertion torque also increased significantly (*p* < 0.001). In turn, the size of the multivariate effect (η*p*^2^ = 0.639) (*p* < 0.001) indicated that the variability detected in the insertion torque and the ISQ, at the same time, was explained by 63.9%, due only to the compression protocol, while the implant diameter explained this variability by 27.0% (η*p*^2^ = 0.270) significantly (*p* < 0.001), and the implant length only significantly explained this variability by 12.1% (η*p*^2^ = 0.121) (*p* = 0.030). In addition, according to the univariate analysis, as the compression protocol increased, the ISQ decreased, although not significantly (*p* = 0.166) (Table 2).

Finally, no interaction between the compression protocol, implant diameter, and length was shown to be an influencing factor in insertion torque variability or the ISQ (*p* > 0.05) (Table 2).

## 4. Discussion

It is known that to achieve good primary stability, there are variables that directly influence this, such as the macrodesign of dental implants (DIs) and bone quality, but there are other variables as important as the surgical drilling protocol that can directly affect the ITVs and ISQ values [3,11]. In the present study, the macrodesign of dental implants and bone density were standardized in order to evaluate the influence of the compressive surgical protocol, considering various lengths and diameters of dental implants, on their primary stability. Although in vitro bone density can be assessed objectively, specialists rely on CBCT scans and their tactile sense to estimate bone quality. This estimate is usually made during initial site preparation and is based on the resistance felt during drilling. Rosas et al. [3] reported that implant length, implant diameter, or surgical undersized protocols could influence the primary stability of the implant by increasing the ITV. To confirm this statement, it is necessary to perform a multivariate analysis, since by its nature, it allows for more exact estimates when more than one effect is measured, since to measure primary stability with the ITV and ISQ, it is necessary to evaluate the effects produced by bone quality, implant length, implant diameter, and surgical protocol at the same time and not separately. For this reason, the purpose of this study was to evaluate primary stability by means of the insertion torque value (ITV) and resonance frequency analysis (RFA) of dental implants placed in standardized blocks of bone quality equivalent to a type II-A bone using three surgical under-milling protocols of 0.2 mm, 0.5 mm, and 0.8 mm, considering different diameters and lengths of the DI. When analyzing the results of the ITV and ISQ, the null hypothesis was rejected.

The introduction and widespread use of the immediate loading technique is of increasing interest in strategies to improve primary implant stability, specifically in low-density bone sites, where specialists need a reliable way to achieve better primary stability of the DI. Although several surgical techniques have been proposed to improve implant stabilization in low-density bone [22], the most promising and simple approach seems to be to perform under-milling at the surgical site [23]. However, it would be crucial to quantitatively determine the amount of under-milling necessary to improve the primary stability of the implant without putting it at risk of additional clinical complications. Regardless of the surgical technique used or the macrodesign of the implant, the percentage reported in the literature regarding the decrease in the prepared bone bed in relation to the diameter of the implant used is very variable [24]. The present study examined three different levels of under-milling (0.2, 0.5, and 0.8 mm). The results of the multivariate analysis showed that the significantly highest average ITV was obtained with a compressive protocol of 0.8 (63.2 ± 14.9 Ncm) (*p* < 0.001), regardless of the length or diameter of the dental implant used. On the other hand, the significantly lowest ITV was obtained with an undersized surgical protocol of 0.2 (25.1 ± 8.3 Ncm) (*p* < 0.001). This result is inconsistent with the study by Degidi et al. [25], who reported that in the presence of poor-quality bone, a 10% undersized protocol equivalent to our study’s 0.2 mm protocol was sufficient to improve the primary stability of an implant, since further decreases did not seem to improve the primary stability values. This difference may be due to the sample used, which comprised fresh, moist bovine bone, classified as type IV bone according to Lekholm and Zarb. [26], while in the present study, the blocks were type II-A. On the other hand, the results obtained were similar to those reported by Tabassumet al. in an in vitro study using polyurethane blocks [27], in which 10 etched tapered implants inserted with a standard protocol produced a mean maximum ITV of 37.2 ± 5.1 Ncm, whereas a similar number of implants with a 14% under-milling protocol produced a mean maximum ITV of 54.3 ± 5.3 Ncm, with statistical differences. Interestingly, the same article reported no statistical difference with polyurethane blocks simulating a denser bone.

Ahn et al. [28] performed another in vitro study, where they used polyurethane blocks adding 1 mm of short epoxy fiber-filled sheet to simulate the cortex. The results showed that the adoption of a 6% under-milling protocol, equivalent in our study to the 0.2 mm protocol, statistically increased primary stability. The maximum ITVs were 104.57 ± 18.16 Ncm in the undersized protocol, which were 25.05 ± 8.24 Ncm in our study. The high values found in the study by Ahn et al. are probably due to the presence of an artificial cortical layer in the polyurethane blocks, since in their study, the implants were placed in a crestal position and being at the level of the cortex, the torque values were considerably elevated. This possibly produced a measurement bias, since the cone morse implants must be placed at a subcrestal level, that is, below the cortical bone, which cancels out the influence of the cortex. In the present study, blocks that considered only the cancellous bone were used, since the primary stability of the cone morse implants is mainly given by the influence of the cancellous bone, this model being closer to the patients’ bone.

In this study, it was found that the degree of bone compression according to the surgical protocol used significantly influenced primary stability, which is consistent with what is reported by Lemos et al. [29] in fresh bovine patellas, which were classified as type III bone in terms of bone density according to the Lekholm and Zarb classification; four different types of implant site preparations were performed, one being the control group and the others being different variations in implant undersized protocols. Furthermore, when evaluating the mean RFA (ISQ) values for the 3.5 and 4.0 mm implant diameters, the stability values were higher when subpreparation was performed than when the sequence proposed by the manufacturer was used. When the RFA measurements were analyzed independently, higher ISQ values were observed for the 3.5 mm diameter implants in the subpreparation sequences that did not use cortical milling. A similar situation was found when analyzing the 4 mm diameter implants, although the differences between the different preparation sequences were not significant. Likewise, the ITVs showed a tendency to increase when there was a reduction in the drill sequence of the preparation technique, which was consistent with our study. Furthermore, in the study conducted by Farronato et al. [30] in polyurethane blocks in which an under-milling protocol was used, an increase in the values for both the ITV and RFA was reported when compared with the implants placed with the conventional protocol, the results obtained being consistent with our studies.

In 2018, a systematic review concluded that although there may be a correlation between torque and ISQ values, they are independent methods for measuring primary stability and comparisons with each other are not recommended [20]. This is because both measure different aspects, since the insertion torque only evaluates the mechanical resistance of the bone to the rotational insertion of the implant, while the ISQ evaluates the stiffness of the bone–implant contact and therefore its resistance to lateral displacement. For this reason, in the present study, it was not sought to establish a correlation between the ITV and the RFA. In this study, when the size of the effect was evaluated, it was evident that when the surgical protocol is optimal according to the bone bed, regardless of the diameter (3.5, 3.8, 4.5, and 5.0 mm) or the length (8.5, 10.0, 11.5, 13.0, and 15.0 mm), primary stability is ensured according to the ITV and RFA by 63.9%. In turn, if the DI diameter is optimal according to the bone bed, regardless of the surgical protocol (0.2 mm, 0.5 mm, and 0.8 mm) or the length (8.5, 10.0, 11.5, 13.0, and 15.0 mm), primary stability is ensured according to the ITV and RFA by 27.0%. Finally, if the DI length is optimal according to the bone bed, regardless of the surgical protocol (0.2, 0.5, and 0.8 mm) or the diameter (3.5, 3.8, 4.5, and 5.0 mm), primary stability is ensured according to the ITV and RFA by 12.1%. This shows that emphasis should be given to the surgical protocol, since it showed a higher percentage of success, which is consistent with the study by Ahn et al. [28], because it reported in the standard surgical protocol an RFA value of 63.30 ISQ while in the undersized, it was 66.50 ISQ.

On the other hand, this study showed that when evaluating the interaction of the surgical protocol versus the diameter and length of the DI, no significant effect was found on primary stability, which could indicate that these variables directly influence primary stability independently without the others affecting their performance. This is consistent with the study by Degidi et al. [25]. In his study, he also found no relationship between the increase in diameter and the increase in RFA according to surgical protocol; he found an RFA value of 69.0, 73.0, and 72.0 ISQ for implants of 3.4, 3.8, and 4.5 mm, respectively, and no statistically significant differences were found.

Among the complications to achieve primary stability in the under-milling protocol, we can include the lack of settlement of six dental implants, since they reached 80 Ncm and could not settle completely. The first of them (protocol of 0.5 mm, 4.5 mm × 15 mm), the second (protocol of 0.8 mm, 3.5 × 15 mm), the third (protocol of 0.8 mm, 4.5 mm × 11.5 mm), the fourth (protocol of 0.8 mm, 4.5 mm × 13 m), the fifth (protocol of 0.8 mm, 5.0 mm × 8.5 mm), and finally, the sixth (protocol of 0.8 mm, 5 × 11.5 mm) were tested. This is in line with what was mentioned above about the influence of the surgical protocol, since in most cases where the implant positioning was not achieved, it was due to the use of the extreme undersized protocol, which was 0.8 mm. On the other hand, five implants presented a risk in primary stability reaching values less than 15 Ncm, the first of them being (0.2 mm protocol, 3.5 × 11.5 mm), the second (0.2 mm protocol, 3.8 × 11.5 mm), the third (0.2 mm protocol, diameter of 4.5 × 10 mm), the fourth (0.2 mm protocol, 4.5 × 11.5 mm), and finally, the fifth (0.2 mm protocol, 4.5 × 13 mm). In this way, it can be seen how the over-milling surgical protocol directly influences the primary stability of dental implants.

It should be kept in mind that the association of more or less compressive surgical protocols with different qualities of cancellous bone could directly affect the primary stability of dental implants. In the present study, it was observed that protocols of 0.8 mm in bones of greater bone density do not allow correct seating of the dental implant, obtaining torque values of more than 80 Ncm, which could alter the macrogeometry of the implant connection or produce compression osteitis in the patient accompanied by pain for several days with a consequent loss of the dental implant. Protocols of 0.2 mm in bones with low bone density gave us torque values well below 15 Ncm, which could clinically translate into a loss of the dental implant in the process of secondary stability due to bone remodeling by osteoclastic action. It is therefore recommended that the surgical protocol for subpreparation be appropriately selected according to bone quality.

Since the above-mentioned studies [25,27] evaluated varying undersized percentages, it is difficult to compare our findings with those of other studies with a similar approach. Although this study showed a significant influence of the ITV and RFA when the compressive insertion protocol, diameter, and length were varied separately, additional in vitro and in vivo studies that include different implant designs, cortical bone thickness, and cancellous bone quality are still needed to investigate the best surgical protocols to ensure primary stability and the final results of implant treatment. To determine appropriate ITVs or ISQ values, it is recommended that clinical studies include other variables such as the crestal or subcrestal position of the dental implant, cortical bone thickness, the presence of different cancellous bone qualities, or an expanded dental implant platform versus straight platform, among others that could directly influence the primary stability of the dental implant. In addition, it should be noted that ITVs are not comparable to ISQ values, as they are two different measures, the former measuring the mechanical locking of the dental implant and the latter measuring the degree of vibration of the dental implant according to the degree of locking of the dental implant in the bone.

Bone quality is determined by the cumulative effect of four primary variables, namely the thickness of the cortical, the quantity of cancellous bone, the thickness of the bony trabeculae, and the dimensions of the medullary spaces. It is only possible for bones with a substantial cortical component, such as types I and II, to have an average cortical thickness of 2 mm; other bone types are not capable of achieving this. The implants utilized in the present study represent one of the most commercially available implant types globally. These are the conomorse platform switching implants, which must be placed 2 mm subcrestal (without cortical influence). In contrast, the external hexagon or internal connection implants represent a minority and are placed at the crestal level (with cortical influence). Accordingly, to more closely approximate a genuine scenario, it was deemed appropriate to utilize models devoid of cortical bone, thereby more accurately simulating the bone stability derived exclusively from cancellous bone, without the confounding influence of cortical bone in the simulation model. In contrast, in bones of poor quality, such as types III-A, III-B, or IV [7], the thickness of the cortical bone does not exceed one millimeter. Consequently, when implants are placed subcrestally, as with the conomorse switching platform, the influence of the cortical bone is negated. Therefore, in our study, the decision was made to use bone models without cortical bone.

It must be recognized that the use of synthetic bone models does not fully reproduce the conditions of human bone. However, it is necessary to start from a standardization of bone quality to minimize the variability of the results and to be able to better observe the influence of the surgical protocol on the primary stability of dental implants. On the other hand, there is no human model with a standardized microarchitecture of the bone trabeculae or the size of the medullary spaces, so the use of a synthetic bone model would be justified for the present study, as it has a more standardized cancellous structure.

The present study evaluated the primary stability or mechanical locking stability of dental implants at the precise moment of placement within the bone. Therefore, further studies are recommended to evaluate the influence of various surgical protocols on the secondary stability or long-term stability leading to the osseointegration of dental implants. However, it should be noted that longitudinal studies in polyurethane models are not feasible due to the biological component of long-term stability, which involves bone remodeling cells and in which factors such as age, endocrine status, and the presence or absence of systemic diseases play an important role.

## 5. Conclusions

Recognizing the limitations of this in vitro study, it can be concluded that when the surgical subpreparation protocol is optimal in terms of the prepared bone bed, regardless of the diameter or length of the dental implant used, primary stability according to the MOT and RFA was assured by 63.9%, which allows us to recommend carrying out a correct analysis of bone quality to subsequently select the most appropriate surgical subpreparation protocol for the bone bed in order to achieve better primary stability of the dental implant. Likewise, we recommend using surgical subpreparation protocols greater than 0.5 mm between the bone bed and the diameter of the dental implant in bones of poor bone quality and protocols less than 0.5 mm in very compact bones, which could be beneficial when an average ITV of 30 to 50 Ncm is required.

## Figures and Tables

**Table 1:** Description and comparison of primary stability using ITV (Ncm) and RFA (ISQ), according to the compressive protocol used.

Dependent Variable	Protocol	n	Mean	SD	SE	95% CI		* *p*	Protocol	
						LL	UL		0.5	0.8
**ITV (Ncm)**	0.2	40	25.05	8.34	1.32	22.38	27.72	<0.001 *	** *p* < 0.001	** *p* < 0.001
	0.5	40	47.33	14.22	2.25	42.78	51.87			** *p* < 0.001
	0.8	40	63.23	14.90	2.36	58.46	67.99			
**RFA (ISQ)**	0.2	40	67.55	5.39	0.85	65.83	69.27	0.166		
	0.5	40	65.78	3.37	0.53	64.70	66.85			
	0.8	40	65.68	4.02	0.64	64.39	66.96			

n: sample; ITV: insertion torque value; (N): Newton; RFA: resonance frequency analysis; ISQ: implant stability quotient; SD: standard deviation; SE: standard error of the mean; 95% CI: 95% confidence interval; LL: lower limit; UL: upper limit. * based on Welch’s one-way intergroup robust ANOVA (* *p* < 0.05, significant differences); ** based on Tukey’s post hoc test (** *p* < 0.05, significant differences).

**Table 2:** Multivariate analysis of primary stability using ITV (Ncm) and RFA (ISQ), according to the compressive protocol used, the diameter, the length of the implant, and their interactions.

Cause	Dependent Variable	Significance and Effect Size				
		Univariate		Multivariate		
		*p* *	η*p*^2^	Value	*p* **	η*p*^2^
**Protocol**	ITV (Ncm)	<0.001 *	0.762	0.130	<0.001 **	0.639
	ISQ	0.166	0.088			
**Diameter**	ITV	<0.001 *	0.327	0.532	<0.001 **	0.270
	ISQ	<0.001 *	0.417			
**Length**	ITV	0.030 *	0.161	0.773	0.048 **	0.121
	ISQ	0.103	0.119			
**Protocol × Diameter**	ITV	0.470	0.086	0.768	0.182	0.124
	ISQ	0.915	0.033			
**Protocol × Length**	ITV	0.975	0.034	0.755	0.353	0.131
	ISQ	0.560	0.102			
**Diameter × Length**	ITV	0.528	0.156	0.613	0.140	0.217
	ISQ	0.528	0.156			
**Protocol × Diameter × Length**	ITV	1000	0.091	0.636	0.967	0.203
	ISQ	0.959	0.173			
**Corrected model**	ITV (a)	<0.001 *	0.811			
	ISQ (b)	0.056	0.598			

ITV: insertion torque value; (N): Newton; RFA: resonance frequency analysis; ISQ: implant stability quotient. * based on Welch’s one-way intergroup robust ANOVA (* *p* < 0.05, significant differences); ** based on Wilks’ Lambda MANOVA with two dependent variables (** *p* < 0.05, significant differences); η*p*^2^: effect size based on partial Eta squared. (a) R^2^ = 0.811 (adjusted R^2^ = 0.625), (b) R^2^ = 0.598 (adjusted R^2^ = 0.203).

## Data Availability

The original contributions presented in this study are included in the article. Further inquiries can be directed to the corresponding authors.
